# Neogenin recruitment of the WAVE regulatory complex maintains adherens junction stability and tension

**DOI:** 10.1038/ncomms11082

**Published:** 2016-03-31

**Authors:** Natalie K. Lee, Ka Wai Fok, Amanda White, Nicole H. Wilson, Conor J. O'Leary, Hayley L. Cox, Magdalene Michael, Alpha S. Yap, Helen M. Cooper

**Affiliations:** 1 Queensland Brain Institute, The University of Queensland, Brisbane, Queensland 4072, Australia; 2 Institute for Molecular Bioscience, The University of Queensland, Brisbane, Queensland 4072, Australia

## Abstract

To maintain tissue integrity during epithelial morphogenesis, adherens junctions (AJs) must resist the mechanical stresses exerted by dynamic tissue movements. Junctional stability is dependent on actomyosin contractility within the actin ring. Here we describe a novel function for the axon guidance receptor, Neogenin, as a key component of the actin nucleation machinery governing junctional stability. Loss of Neogenin perturbs AJs and attenuates junctional tension. Neogenin promotes actin nucleation at AJs by recruiting the Wave regulatory complex (WRC) and Arp2/3. A direct interaction between the Neogenin WIRS domain and the WRC is crucial for the spatially restricted recruitment of the WRC to the junction. Thus, we provide the first example of a functional WIRS–WRC interaction in epithelia. We further show that Neogenin regulates cadherin recycling at the AJ. In summary, we identify Neogenin as a pivotal component of the AJ, where it influences both cadherin dynamics and junctional tension.

## Abstract (web summary)

The stability of epithelial adherens junctions depends on tension generated by actomyosin contractility. Here Lee *et al*. describe a novel role for the axon guidance receptor Neogenin in maintaining junctional stability by recruiting actin nucleation machinery to adherens junctions.

## Graphical abstract

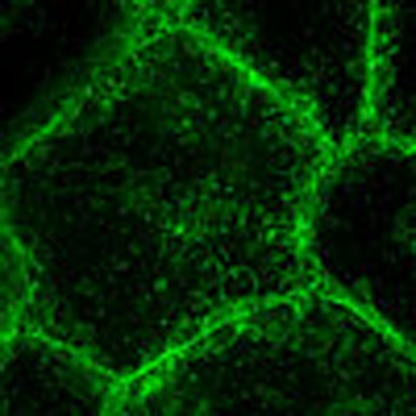

Epithelial morphogenesis is a fundamental process driving organogenesis in the embryo. Simple epithelial sheets undergo choreographed movements to generate complex structures such as the epithelial tube of the gut and the neural tube, the precursor of the brain and spinal cord. Dynamic cellular behaviours including epithelial folding and lumen formation require that epithelial sheets remain flexible but resilient in response to local stresses^1^. E-cadherin (Ecad)-mediated cell–cell adhesion found at adherens junctions (AJs) plays a key role in maintaining the fidelity of the epithelium. Junctional stability entails reciprocal interactions between the cadherins and the circumferential actin ring running parallel to the AJ^2^,^3^,^4^,^5^,^6^. A primary function of the actin ring is to regulate junctional tension through actomyosin contractility^7^,^8^. Critically, contractility at the AJ not only mitigates against external forces but also actively drives cell movements by exerting contractile tension on adjacent cells^3^,^9^,^10^. The actin ring undergoes continual turnover^4^,^11^, and failure to rebuild this network results in loss of adhesion, AJ disruption and epithelial disintegration^12^,^13^,^14^. The molecular pathways that promote actin nucleation strictly within the spatial confines of the AJ are therefore crucial factors safeguarding the fidelity of organogenesis and nervous system development.

The actin ring is maintained by Arp2/3 nucleation at the AJ^11^,^15^,^16^. Tight spatiotemporal control of Arp2/3 activity is coordinated through its association with the WAVE regulatory complex (WRC), comprising five subunits organized into the Sra/Nap and WAVE/Abi/HSPC300 subcomplexes^17^,^18^,^19^. The WRC exists in an inactive form in which the Arp2/3-binding domain of WAVE (the VCA domain) is sequestered^20^,^21^. Binding of activated Rac to the Sra subunit induces a conformational change within the complex, leading to the release of the VCA domain^22^. Currently, the mechanism by which the actin nucleation machinery is recruited to the cadherin complex is poorly understood. We now show that Neogenin, a member of the deleted in colorectal cancer (DCC) guidance receptor family^23^,^24^, is an essential junctional component where it promotes actin ring stability by spatially coupling Arp2/3-mediated actin nucleation at the AJ via a direct interaction with the WRC.

Neogenin is a receptor for both Netrin-1 and the repulsive guidance molecules (RGMs), and is essential for key embryonic processes, including myogenesis, chondrogenesis and neural tube formation^25^,^26^,^27^,^28^. Its function is best understood in the nervous system, where it controls cell and axon migration during embryogenesis and neural progenitor migration and cell cycle kinetics in the adult^29^,^30^,^31^,^32^,^33^,^34^,^35^. Depletion of Neogenin in the neuroepithelium of the early neural tube leads to loss of adhesion, the inability to establish apicobasal polarity and ultimately a failure in lumen formation^25^,^26^,^36^. Here we report that Neogenin is also found in simple epithelia such as the colonic cell line Caco-2, a well-established *in vitro* epithelial model for the study of junction formation^17^,^37^,^38^,^39^. We demonstrate that Neogenin is pivotal to the maintenance of junctional stability by regulating Ecad recycling and modulating junctional tension via WRC/Arp2/3-mediated actin nucleation.

## Results

### Neogenin controls AJ stability but not assembly

**Figure 1:**
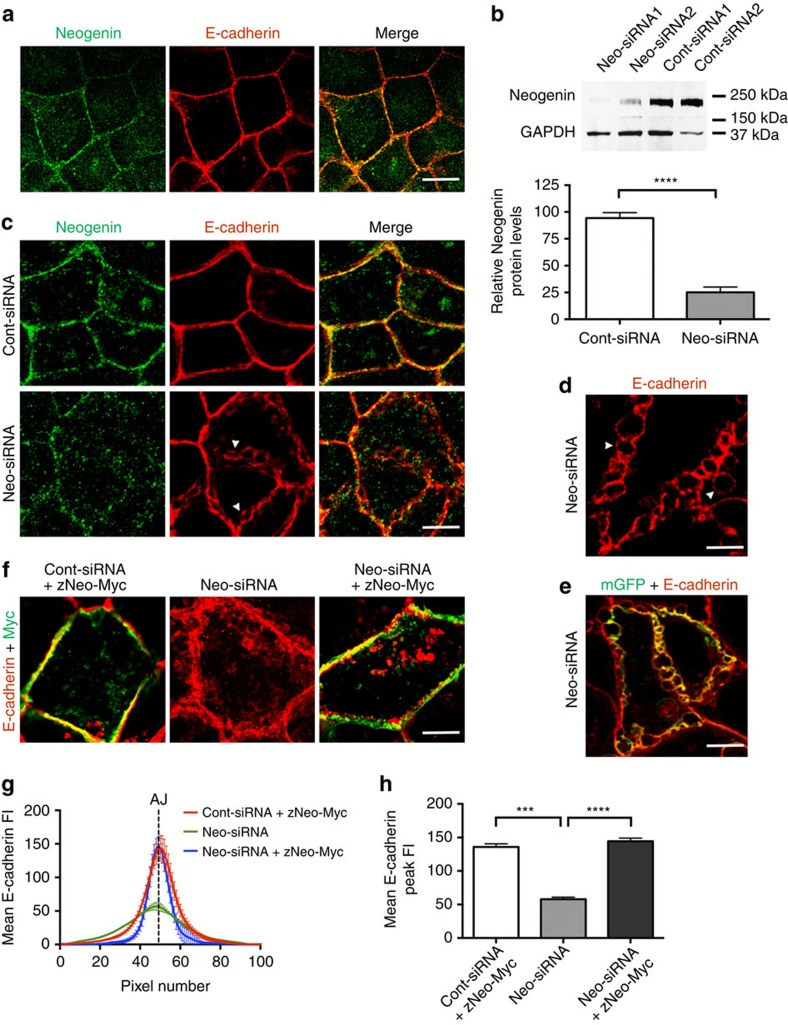
Neogenin maintains AJ integrity. (**a**) Representative confocal micrograph showing apical view of Caco-2 cells where Neogenin (green) co-localized with Ecad (red; merge, yellow) at the AJ. (**b**) Immunoblot densitometry of Neogenin protein levels in Neo-siRNA cells relative to the control protein, GAPDH (*n*=3, mean±s.e.m., ****P<0.0001, Student's *t*-test). (**c**) Neo-siRNA induced the loss of adhesion between Ecad+ membranes, leading to the formation of tightly packed bleb-like structures at the AJ (arrowheads; Ecad, red; Neogenin, green). (**d**) Higher-magnification image of disrupted AJs in Neo-siRNA-expressing cells. (**e**) Co-localization of membrane-bound GFP (mGFP, green) and Ecad (red; merge, yellow) in Neo-siRNA-transfected cells. (**f**) Full-length zebrafish Neogenin (zNeo-Myc) rescued junctional integrity (Myc, green; Ecad, red). Line-scan analysis: (**g**) mean Ecad fluorescence intensity (FI) across AJs. (**h**) Mean Ecad peak FI at the AJ (*n*=150 junctions, mean±s.e.m., ***P<0.001, ****P<0.0001, one-way analysis of variance (ANOVA), Dunn's *post hoc* test). Scale bars, 15 μm (**a**,**c**); 5 μm (**d**,**e**); 7 μm (**f**).

Immunolabelling of polarized Caco-2 monolayers demonstrated that Neogenin co-localizes with Ecad at the AJ (Fig. 1a), suggesting that it may participate in junction assembly or maintenance. To investigate the role of Neogenin at the AJ, we used small-interfering RNAs (Neo-siRNAs) to knockdown Neogenin. Neogenin was reduced by 75% in Neo-siRNA cells, and immunolabelling of confluent monolayers 2 days post transfection revealed substantial depletion of Neogenin from the AJ (Fig. 1b,c). Equivalent phenotypes were observed for two independent Neo-siRNA sequences (Supplementary Fig. 1b). Apical views of the AJ identified by Ecad immunoreactivity showed that the plasma membranes from adjacent cells were tightly apposed in cells transfected with control siRNA (Cont-siRNA; Fig. 1c). In striking contrast, substantial disruption of the AJ was observed in Neo-siRNA cells, where the linear Ecad-positive (+) junction was replaced by bleb-like structures (Fig. 1c,d). Cotransfection of a membrane-bound GFP (mGFP) and Neo-siRNA demonstrated that Ecad co-localized with mGFP in these structures, confirming junctional disruption (Fig. 1e). Despite the marked AJ phenotype, localization of the tight junction markers ZO-1 and aPKC was unaffected (Supplementary Fig. 1c,d), indicating that apicobasal polarization and tight junction formation were not perturbed. Line-scan analysis of Ecad fluorescence intensity across the AJ confirmed the loss of junctional organization as the average pixel intensity at the AJ was reduced 2.5-fold after Neogenin depletion (Fig. 1g,h). Finally, coexpression of zebrafish Neogenin (zNeo-Myc) fully restored AJ integrity (Fig. 1f–h), demonstrating that this phenotype was Neogenin-specific.

**Figure 2:**
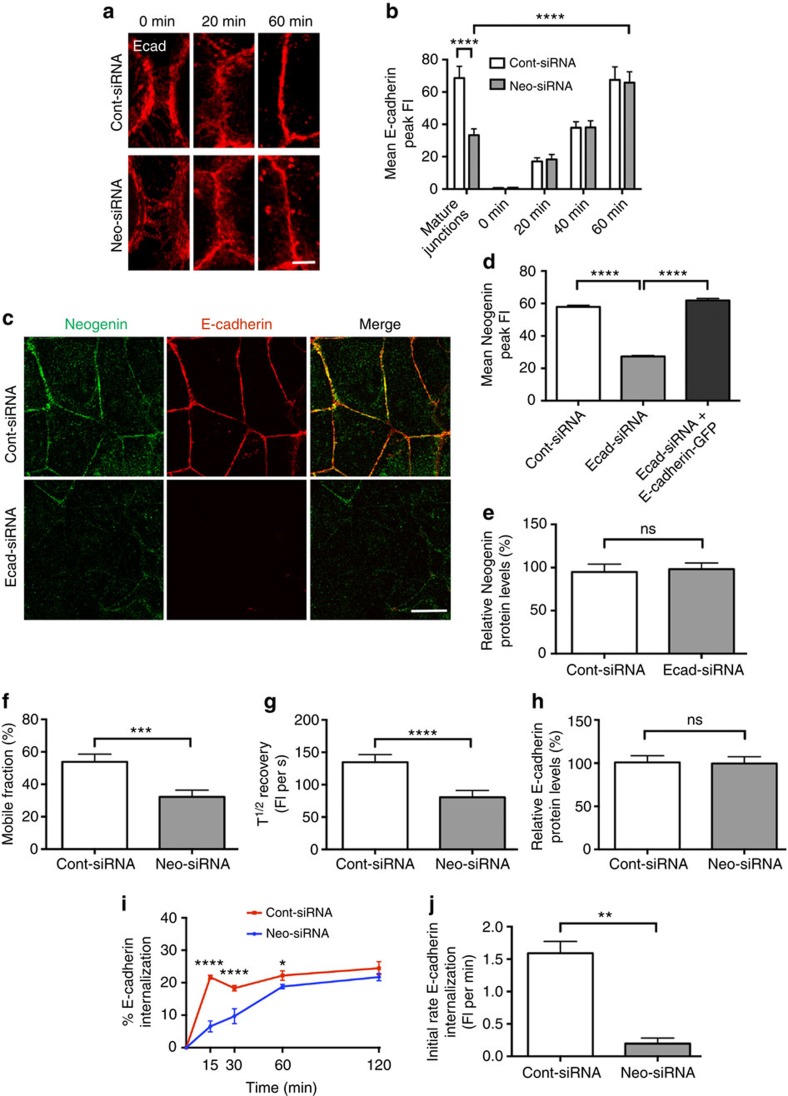
Neogenin controls cadherin recycling and AJ stability but not junction assembly. (**a**,**b**) Calcium depletion assays demonstrated that Neogenin was not required for junction assembly. (**a**) Re-establishment of AJs (Ecad, red) 0–60 min after calcium replacement. (**b**) Line-scan analysis: mean Ecad peak fluorescence intensity (FI) 0–60 min after calcium replacement (*n*=150 junctions, ****P<0.0001, two-way analysis of variance (ANOVA), Bonferroni's *post hoc* test). (**c**) Neogenin (green) was lost from the AJ after transfection of Ecad-siRNA (Ecad, red). (**d**) Line-scan analysis shows that coexpression of Ecad-GFP with Ecad-siRNA rescued Neogenin levels at the AJ: mean Neogenin peak FI at the AJ (*n*=150 junctions, mean±s.e.m., ****P<0.0001, Student's *t*-test). (**e**) Densitometry of immunoblots: total cellular Neogenin levels were equivalent in the presence and absence of Ecad (*n*=3, mean±s.e.m., *P*>0.05, Student's *t*-test). (**f**,**g**) FRAP analysis revealed that loss of Neogenin reduced the mobile pool of Ecad-GFP (**f**) and the time taken to reach 50% maximum fluorescence recovery (T^1/2^ recovery) after bleaching (**g**) (*n*>30, mean±s.e.m., ***P<0.001, ****P<0.0001, Student's *t*-test). (**h**) Densitometry of immunoblots: total cellular Ecad levels were equivalent in the presence and absence of Neogenin (*n*=3, mean±s.e.m., *P*>0.05, Student's *t*-test). (**i**) Surface biotinylated-Ecad internalization was impeded in Neo-siRNA cells after raising the temperature from 0 to 37 °C (*n*=3, mean±s.e.m., ****P<0.0001, *P<0.05, two-way ANOVA, Holm's multiple comparison test). (**j**) The initial rate (0–15 min) of biotinylated-Ecad internalization was significantly reduced (*n*=3, mean±s.e.m., **P<0.01, Student's *t*-test). ns, not significant. Scale bars, 3 μm (**a**); 15 μm (**c**).

AJ perturbation resulting from loss of Neogenin may be due to the inability of cadherins to undergo adhesive homophilic interactions at the cell–cell interface and thus initiate junction formation. To investigate this, we performed calcium depletion assays in which cadherin adhesion was disrupted by the addition of the calcium chelator EGTA^40^,^41^. Nascent cadherin-mediated cell–cell contacts were re-established between control cells 20 min after calcium replenishment, and AJ assembly was complete by 60 min (Fig. 2a,b). Neogenin-depleted cells also re-initiated new contacts within 20 min, and tight Ecad+ junctions were observed between cells after 60 min, indicating that junctions formed normally. Line-scan analysis confirmed that the accumulation of cadherin at immature junctions and the rate of junctional assembly did not depend on Neogenin (Fig. 2b). Therefore, Neogenin is not required for Ecad-mediated AJ assembly and maturation.

To further explore the relationship between Neogenin and Ecad we asked whether the junctional localization of Neogenin might depend on Ecad. As expected, siRNA knockdown of Ecad (Supplementary Fig. 2a) resulted in loss of adhesion at the AJ^17^,^38^. Moreover, line-scan analysis revealed a significant depletion of Neogenin from the junctional interface (Fig. 2c,d). However, total cellular Neogenin levels remained constant in the absence of Ecad (Fig. 2e). Junctional localization of Neogenin was fully restored when Ecad-GFP was cotransfected with the Ecad-siRNA (Fig. 2d). Therefore, localization of Neogenin at the mature AJ requires Ecad.

### Neogenin controls Ecad recycling at the AJ

Although the steady-state levels of Ecad on the plasma membrane appeared unaffected in Neogenin-depleted cells (Figs 1c, 2b), it was possible that AJ disruption was due to a perturbation in Ecad recycling, an important contributor to junctional integrity^42^,^43^. FRAP (fluorescence recovery after photobleaching) analysis^17^,^42^,^44^ revealed a 34% decrease in the size of the mobile Ecad pool and a 33% reduction in fluorescence recovery at Neo-siRNA junctions (Fig. 2f,g), indicating that Ecad dynamics at the junctional membrane were altered. However, this effect did not result from loss of total cellular Ecad levels (Fig. 2h). We next assessed whether Ecad endocytosis was impeded in Neo-siRNA cells using a previously described internalization assay, where cadherins were surface-biotinylated on ice (to block endocytosis), and the uptake of labelled cadherin was then measured after the temperature block was released^44^. In the absence of Neogenin we observed a 2.0- to 3.3-fold decrease in internalized Ecad after 15–30 min and a 3.4-fold reduction in the initial rate of Ecad internalization (Fig. 2i,j), indicating that Neogenin was required for Ecad endocytosis. All internalization was prevented in the presence of chlorpromazine, which can inhibit clathrin-mediated endocytosis (Supplementary Fig. 2b). Therefore, suppression of Ecad recycling within the junctional membrane may contribute, at last in part, to the junctional defect in Neo-siRNA cells.

### Actin nucleation and AJ tension requires Neogenin

**Figure 3:**
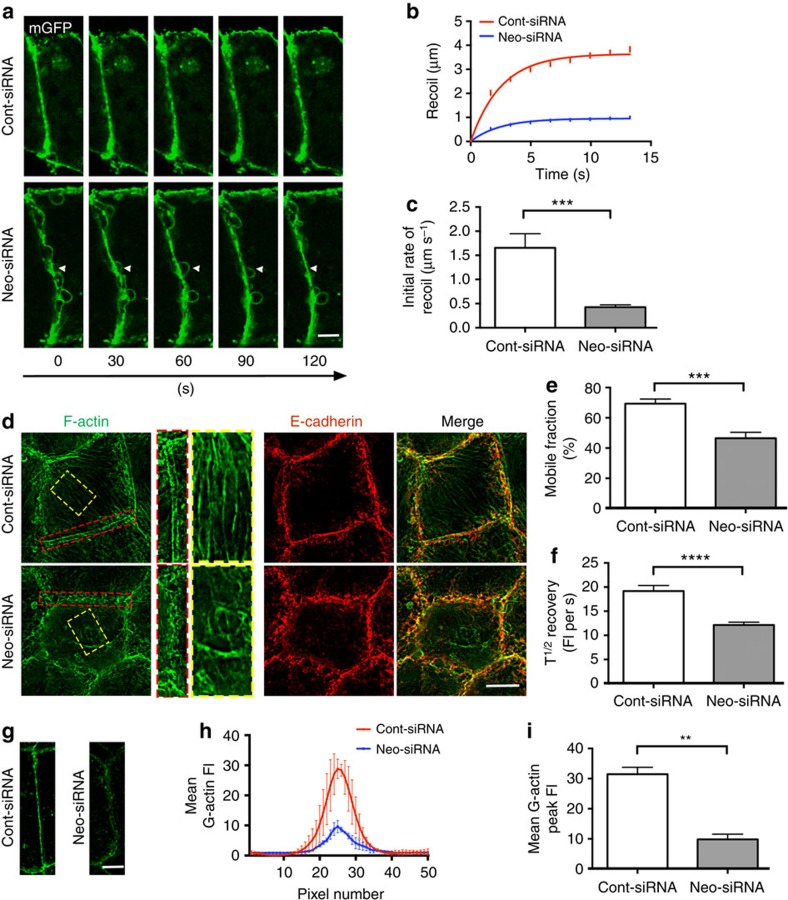
Junctional tension is mediated by Neogenin-dependent perijunctional actin nucleation. (**a**) Time-lapse confocal micrographs (0–120 s) of AJs in Caco-2 cells expressing mGFP (green) and Neo-siRNA (membrane protrusions, arrowheads). (**b**,**c**) Junctional tension was quantified using the laser nanoscissors technique. Loss of tension was demonstrated by the reduction in the extent of recoil towards the vertices and the rate of initial membrane recoil (*n*>38 junctions; mean±s.e.m.; ***P<0.001, Student's *t*-test). (**d**) Structured illumination micrographs show disruption of the actin cytoskeleton in Neo-siRNA cells (red box, junctional actin; yellow box, stress fibres; phalloidin, green; Ecad, red). FRAP analysis showed that loss of Neogenin reduced the mobile pool of F-actin at the AJ (**e**) and the time taken to reach 50% maximum fluorescence recovery (T^1/2^ recovery) after bleaching (**f**) (*n*>30, mean±s.e.m., ***P<0.001, ****P<0.0001, Student's *t*-test). (**g**) Fewer G-actin+ barbed ends (green) were seen adjacent to the AJ, demonstrating a reduction in actin nucleation in the absence of Neogenin. Line-scan analysis: (**h**) mean G-actin fluorescence intensity (FI) across AJs. (**i**) Mean G-actin peak FI at the AJ (*n*=150 junctions, mean ±s.e.m., **P<0.01, Student's *t*-test). Scale bars, 2 μm (**a**); 7 μm (**d**); 15 μm (**g**).

Time-lapse imaging of Caco-2 monolayers cotransfected with Neo-siRNA and mGFP revealed unusual, dynamic membrane behaviour at the junctional interface. In control cells we observed minor membrane fluctuations at the level of the apical junction (Supplementary Movie 1) as has been previously observed when junctional actomyosin is present^45^. In striking contrast, rapid extrusion and collapse of large mGFP+ bleb-like structures was observed at the junctional interface between Neogenin-depleted cells (Supplementary Movie 2 and Fig. 3a), suggesting that junctional tension was attenuated. To assess junctional tension, we employed two-photon laser nanoscissors to sever the junctions and then determined the instantaneous velocity of initial recoil of the vertices (Supplementary Movies 3 and 4), a measure of junctional tension^17^,^39^. Recoil of vertices was significantly decreased in Neogenin-depleted cells, where the extent and rate of recoil were reduced 4.1-fold and 3.9-fold, respectively (Fig. 3b,c). We conclude that Neogenin is essential for the maintenance of junctional tension.

Contractile tension at AJs is supported by the associated actomyosin apparatus, which requires actin assembly at the junction^46^. As tension is compromised when actin assembly is inhibited^17^, we next tested whether loss of tension was due to the collapse of the actin ring. Phalloidin labelling of F-actin highlighted the actin cables running parallel to the AJ and the cytoplasmic stress fibres in Cont-siRNA cells (Fig. 3d). In the absence of Neogenin, the actin ring and stress fibres had disintegrated (Fig. 3d). Disruption of the actin ring was confirmed by live imaging of LifeAct-tagged F-actin. In control cells the actin rings under adjacent junctions remained stable over 200 s (Supplementary Movie 5), whereas the actin filaments thinned and disappeared (arrow, Supplementary Movie 6) in Neo-siRNA cells and no clearly defined actin rings were visible. The dynamic behaviour of the junctional actin cytoskeleton was then assessed by FRAP after cotransfection with Neo-siRNA and LifeAct-GFP^17^,^45^. The average recovery of fluorescence intensity in the actin ring was 1.8-fold less efficient in Neogenin-depleted cells, and the size of the actin mobile fraction was reduced by 26% (Fig. 3e,f). Neogenin is therefore required for actin turnover. The actin ring is maintained by cortical actin nucleation at the junctional membrane^11^,^39^. Therefore, we quantified the incorporation of fluorescently tagged G-actin into growing actin filaments in live saponin-permeabilized cells^11^. Line-scan analysis revealed a 3-fold reduction in the number of G-actin-labelled barbed ends at the membrane in Neogenin-depleted cells (Fig. 3g–i), implying that actin nucleation was reduced. Taken together the above data suggest that Neogenin-dependent assembly of the actin ring contributes to the maintenance of contractile tension at the AJ.

### Neogenin binds the WRC to recruit Arp2/3 to the AJ

**Figure 4:**
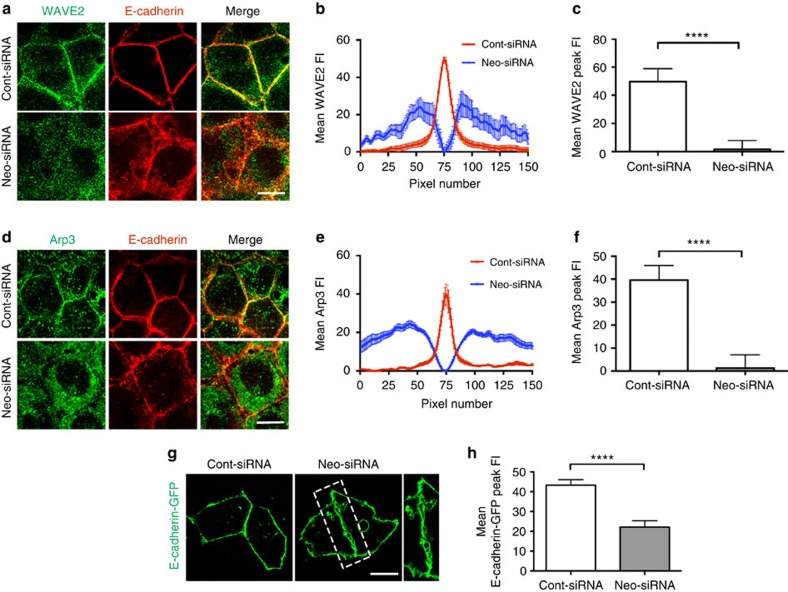
Neogenin recruits WAVE2 and Arp2/3 to the AJ. (**a**) WAVE2 (green) was not associated with Ecad (red) at the AJ in Neo-siRNA-expressing cells. Line-scan analysis: (**b**) mean WAVE2 fluorescence intensity (FI) across AJs and (**c**) mean WAVE2 peak FI at junctions. (**d**) Arp3 (green) was not associated with Ecad (red) at the AJ. Line-scan analysis: (**e**) mean Arp3 FI across AJs and (**f**) mean Arp3 peak FI at junctions. (**g**) Coexpression of full-length Ecad (Ecad-GFP) did not rescue the Neo-siRNA phenotype. (**h**) Line-scan analysis: mean Ecad peak FI in cells coexpressing Neo-siRNA and Ecad-GFP. (**b**,**c**,**e**,**f**,**h**) *n*=150 junctions, mean±s.e.m., ****P<0.0001, Student's *t*-test. Scale bars, 7 μm (**a**,**d**); 15 μm (**g**).

As the actin ring originates from WAVE2–Arp2/3-mediated nucleation at the AJ and as knockdown of either WAVE2 or Arp2/3 reduces junctional tension^17^, we investigated whether the sequestration of Arp3 and the WRC subunit, WAVE2, to junctions requires Neogenin. Line-scan analysis showed that WAVE2 and Arp3 were tightly juxtaposed to Ecad complexes at the AJ in control cells (Fig. 4a–f). Conversely, WAVE2 (Fig. 4a–c) and Arp3 (Fig. 4d–f) were dispersed throughout the cytoplasm in Neo-siRNA cells; however, total cellular WAVE2 and Arp3 levels remained unchanged (Supplementary Fig. 3a,b). These data suggest that Neogenin recruits the WRC and Arp2/3 to the AJ. As the WRC also interacts with Ecad^17^, it was possible that Neogenin was regulating WRC recruitment indirectly. However, cotransfection of full-length Ecad (Ecad-GFP) with Neo-siRNA failed to rescue AJ integrity (Fig. 4g,h), demonstrating that Neogenin functions downstream of Ecad.

**Figure 5:**
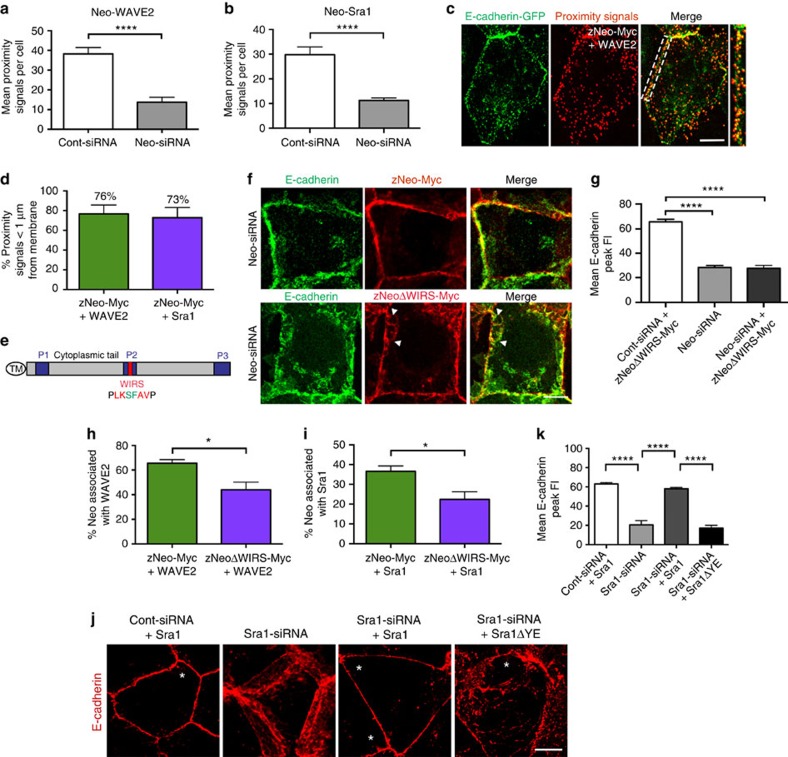
The WIRS domain in Neogenin interacts with the WRC at the AJ. The Duolink proximity assay revealed that Neogenin associated with WAVE2 (**a**) and Sra1 (**b**) in Caco-2 cells (*n*=3 experiments, mean±s.e.m., ****P<0.0001, Student's *t*-test). (**c**) Proximity signals for zNeo-Myc+WAVE2 (red) were closely associated (<1 μm) with the Ecad+ AJ membrane (green). (**d**) Percentage of zNeo-Myc+WAVE2 and zNeo-Myc+Sra1 proximity signals tightly associated (<1 μm) with the AJ relative to total cellular zNeo-Myc+WAVE2 or zNeo-Myc+Sra1 proximity signals, respectively (*n*>27 cells). (**e**) Schematic of Neogenin cytoplasmic domain: WIRS domain (red); mutated amino acids in the WIRS domain (green); and subdomains P1–3 (blue). (**f**) Junctional integrity (Ecad, green) was rescued by zNeo-Myc (upper panel, red) but not by the WIRS mutant (zNeoΔWIRS-Myc, lower panel, red, arrowheads). (**g**) Line-scan analysis: mean Ecad peak fluorescence intensity (FI) in cells expressing Neo-siRNA and zNeoΔWIRS-Myc (*n*=150 junctions, mean±s.e.m., ****P<0.0001, one-way analysis of variance (ANOVA), Dunn's *post hoc* test). (**h**,**i**) The extent of association between zNeoΔWIRS-Myc and WAVE2 or Sra1 was significantly reduced compared with that of zNeo-Myc (*n*>26 cells, mean±s.e.m., *P<0.05, Student's *t*-test). (**j**) Wild-type Sra1 but not Sra1ΔYE rescues AJ integrity in Sra1-siRNA cells (Ecad, red; asterisk represents IRES-GFP+ cell). (**k**) Line-scan analysis: mean Ecad peak FI in cells expressing Sra1-siRNA and Sra1 or Sra1ΔYE (*n*=150 junctions, mean±s.e.m., ****P<0.0001, one-way ANOVA, Dunn's *post hoc* test). Scale bars, 7 μm (**c**,**f**,**j**).

The above data suggest that Neogenin anchors the WRC to the AJ. To assess whether endogenous Neogenin is closely associated with the WRC we employed the Duolink proximity ligation assay, an antibody-based assay that detects protein–protein interactions occurring within 40 nm of each other (see Methods). A significant number of proximity signals per cell was detected for the Neogenin-WAVE2 and -Sra1 antibody pairs in control cells, whereas Neogenin knockdown markedly reduced the proximity signal count (Fig. 5a,b and Supplementary Fig. 4). Thus, there is a close association between endogenous Neogenin and the WRC. The spatial distribution of Neogenin and the WRC relative to the AJ was then determined in wild-type monolayers. To unequivocally demonstrate that Neogenin and the WRC were within nanoscale proximity on the same side of the junctional interface we coexpressed zNeo-Myc with Ecad-GFP. The number of proximity signals derived from Myc-WAVE2 or Myc-Sra1 antibody pairs located within 1 μm of the Ecad+ AJ was then quantified (Fig. 5c,d). This analysis revealed that 73–76% of all zNeo-Myc-WAVE2 and -Sra1 proximity signals were located within 1 μm of the junction. Therefore, we conclude that Neogenin and the WRC are closely associated at the AJ.

The WRC interacting receptor sequence (WIRS) found in WRC-binding partners^47^ is present in the Neogenin cytoplasmic domain (Fig. 5e)^23^. This motif binds to a highly conserved binding pocket within the WRC composed of the Sra1 and Abi subunits, and requires full assembly of the WRC^10^,^21^. To determine if Neogenin recruits the WRC-Arp2/3 complex to the AJ by directly interacting with Sra1 and Abi we mutated the WIRS motif of Neogenin (zNeoΔWIRS-Myc, Fig. 5e), coexpressed zNeoΔWIRS-Myc or zNeo-Myc with Neo-siRNA, and then assessed the junctional integrity by line-scan analysis (Figs 1f–h and 5f,g). zNeo-Myc fully restored Ecad accumulation into AJs. However, as predicted, the WIRS mutant failed to restore junctional integrity. Further analysis revealed that WAVE2 and Arp3 were tightly associated with the cadherin complex at the AJ in control cells, whereas these proteins remained distributed throughout the cytoplasm in Neo-siRNA/zNeoΔWIRS-Myc-expressing cells (Supplementary Fig. 5a,b), indicating that recruitment to cadherin junctions is dependent on the Neogenin–WRC interaction. This interpretation was confirmed by our proximity assay, which revealed a significant reduction in the number of Myc-WAVE2 and -Sra1 proximity signals in cells expressing the WIRS mutant compared with those expressing zNeo-Myc (Fig. 5h,i). Finally, to confirm that the WIRS-binding surface of Sra1 is essential for junctional stability Sra1 siRNAs were cotransfected with Sra1 or a mutant form (Sra1ΔYE) in which residues contributing to the WIRS-binding surface were substituted for alanine^47^. As predicted, knockdown of Sra1 generated the Neogenin phenotype (Fig. 5j,k and Supplementary Fig. 5c). Importantly, whereas wild-type Sra1 fully rescued this phenotype, mutated Sra1 failed to restore junctional integrity. Together, these data clearly demonstrate that a direct Neogenin-Sra1/Abi1 interaction is necessary to recruit the WRC and Arp2/3 to the AJ.

**Figure 6:**
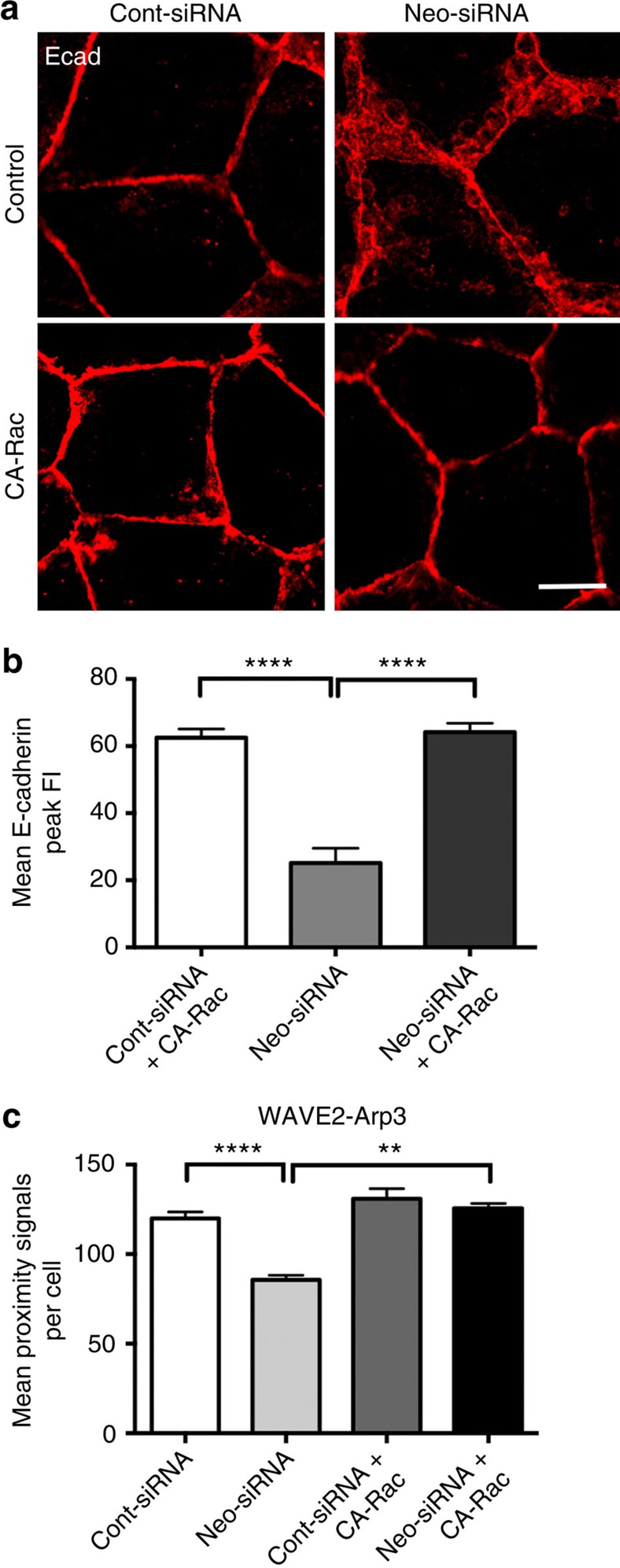
Neogenin requires Rac activation to promote WAVE2–Arp2/3 interactions. (**a**) Cotransfection of CA-Rac with Neo-siRNA rescued junctional integrity. (**b**) Line-scan analysis: mean Ecad peak fluorescence intensity (FI) in cells coexpressing Neo-siRNA and CA-Rac (*n*=150 junctions, mean±s.e.m., ****P<0.0001, one-way analysis of variance (ANOVA), Dunn's *post hoc* test). (**c**) The Duolink proximity assay revealed that WAVE2 associated with Arp3 in cells cotransfected with CA-Rac and Neo-siRNA, but not with Neo-siRNA alone (*n*=3 experiments, mean±s.e.m., **P<0.01, ****P<0.0001, one-way ANOVA, Dunn's *post hoc* test). Scale bar, 15 μm (**a**).

Finally, we asked whether Neogenin-Sra1/Abi binding was sufficient to induce WRC-Arp2/3 association or whether binding of Rac-GTP to Sra1 was also required^21^,^48^. Coexpression of constitutively active Rac (CA-Rac) with Neo-siRNA fully restored AJ integrity (Fig. 6a,b). Furthermore, the Duolink assay revealed a significant reduction (28%) in the number of WAVE2–Arp3 proximity signals per cell in Neo-siRNA cells, whereas CA-Rac restored WAVE2–Arp3 interactions to control levels (Fig. 6c). Therefore, although Neogenin is necessary for the recruitment of the WRC to the AJ, Rac activation is required for WAVE–Arp2/3 interactions.

### Neogenin and RGMa control cyst morphogenesis

**Figure 7:**
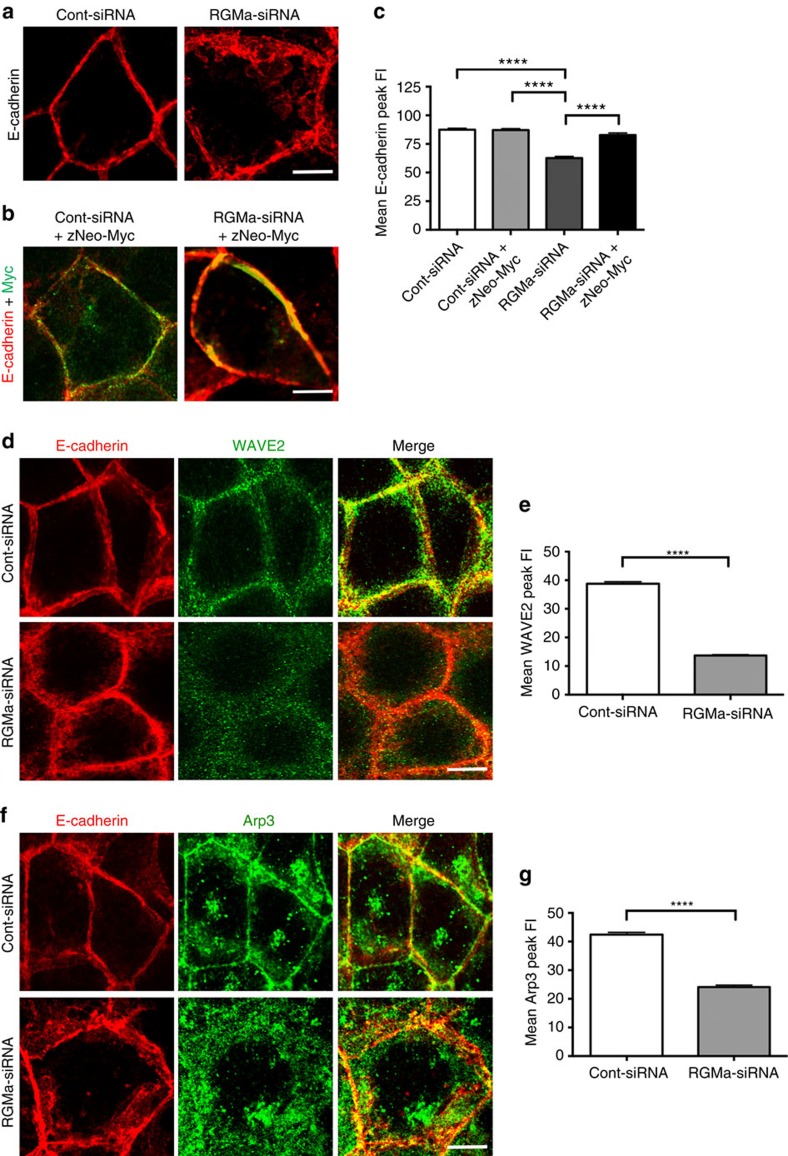
RGMa is required for AJ stability and recruitment of the WRC and Arp2/3. (**a**) RGMa-siRNA induced the loss of junctional integrity (Ecad+, red). (**b**) Coexpression of zNeo-Myc (green; Ecad, red; merge, yellow) with RGMa-siRNA rescued junctional integrity. (**c**) Line-scan analysis: mean Ecad peak fluorescence intensity (FI) in cells coexpressing RGMa-siRNA and zNeo-Myc or RGMa-siRNA alone (*n*=150 junctions, mean±s.e.m., ****P<0.0001, one-way analysis of variance (ANOVA), Dunn's *post hoc* test). (**d**) WAVE2 (green) was not associated with Ecad (red) at the AJ in RGMa-siRNA-expressing cells. (**e**) Line-scan analysis: mean WAVE2 peak FI in RGMa-siRNA cells. (**f**) Arp3 (green) was not associated with Ecad (red) at the AJ in RGMa-siRNA-expressing cells. (**g**) Line-scan analysis: mean Arp3 peak FI in RGMa-siRNA cells. (**e**,**g**) *n*=150 junctions, mean s.e.m., ****P<0.0001, Student's *t*-test. Scale bars, 15 μm (**a**,**b**,**d**,**f**).

As RGMa–Neogenin interactions are essential for cell adhesion in the neuroepithelium of the neural tube^26^ and Netrin-1 is not expressed in Caco-2 cells (Supplementary Fig. 6a), it was likely that RGMa maintained AJ stability in Caco-2 monolayers. siRNA knockdown of RGMa generated an equivalent junctional disruption to that seen in Neo-siRNA cells (Fig. 7a,c and Supplementary Fig. 6b). Colabelling with antibodies to Ecad and WAVE2 or Arp3, followed by line-scan analysis, further revealed that loss of RGMa led to the dissociation of WAVE2 and Arp3 from the AJ (Fig. 7d–g). Moreover, cotransfection of zNeo-Myc with RGMa-siRNA completely restored junctional integrity (Fig. 7b,c), demonstrating that RGMa acts upstream of Neogenin to maintain AJ stability.

**Figure 8:**
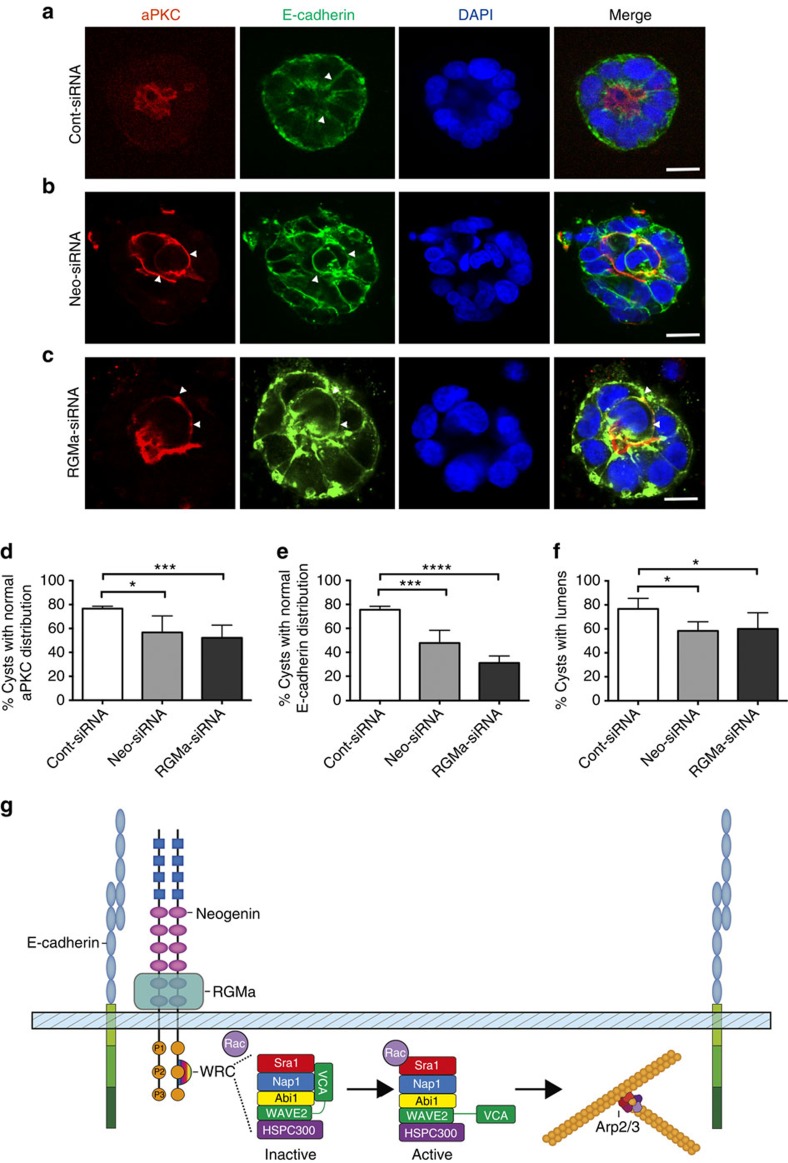
RGMa and Neogenin control Caco-2 epithelial cyst morphogenesis. Representative confocal micrographs of Caco-2 cysts after transfection of (**a**) Cont-siRNA (arrowheads indicate Ecad+ junctions), (**b**) Neo-siRNA or (**c**) RGMa-siRNA. (**b**,**c**) Arrowheads indicate cells that have undergone extrusion. (**d**) Quantification of the number of cysts displaying normal apical aPKC distribution (*n*=30, *P<0.05, ***P=0.001, one-way analysis of variance (ANOVA), Dunn's *post hoc* test). (**e**) Quantification of the number of cysts displaying normal basolateral Ecad distribution (*n*=30, ***P<0.001, ****P<0.0001, one-way ANOVA, Dunn's *post hoc* test). (**f**) Quantification of the number of cysts with lumens (*n*=40, *P<0.05, one-way ANOVA, Dunn's *post hoc* test). (**g**) Proposed model for Neogenin function. Neogenin anchors the WRC at the AJ by promoting Neogenin-WIRS binding to the Sra1 and Abi WRC subunits, allowing Rac-Sra1 binding to facilitate Arp2/3-mediated actin nucleation and preservation of actin contractility. Scale bars, 20 μm (**a**–**c**).

Within a growing three-dimensional epithelial tube, the forces exerted on AJs are considerably more complex than those experienced in a two-dimensional monolayer^49^,^50^. Therefore, we explored the requirement for Neogenin and RGMa in Caco-2 cyst development, a model known to replicate key molecular and cellular events governing epithelial morphogenesis^4^,^39^,^40^,^51^. In mature control cysts, the central lumen was surrounded by an apicobasally polarized epithelium in which individual cells were connected by Ecad+ AJs (Fig. 8a, arrowheads) and aPKC demarcated the apical membrane (Fig. 8a). In contrast, within Neogenin- and RGMa-depleted cysts, cells failed to establish junctions, no longer exhibited a polarized morphology and were extruded into the centre of the cell aggregate (Fig. 8b–e). Consequently, lumen expansion was prevented (Fig. 8f). These data strongly imply that RGMa–Neogenin interactions maintain the integrity of three-dimensional epithelial structures through the modulation of junctional tension.

## Discussion

To maintain tissue integrity during epithelial morphogenesis, adherens junctions must resist the mechanical stresses exerted as a consequence of dynamic cellular behaviours. AJ stabilization is achieved by modulation of actomyosin contractility generated within the actin ring. Here we describe a novel, unexpected function for Neogenin as a key component of the actin nucleation machinery governing AJ stability and tension. To our knowledge this is the first demonstration that an axon guidance receptor can be redeployed to regulate actomyosin contractility at epithelial junctions. We reveal that loss of Neogenin severely perturbs cell adhesion in Caco-2 monolayers accompanied by a substantial attenuation in junctional tension resulting from fragmentation of the actin ring. We demonstrate that Neogenin promotes the formation of stable actin rings at AJs by spatially coupling Arp2/3-mediated actin nucleation to the AJ via the recruitment of the WRC. A direct interaction between the Neogenin WIRS domain and the WRC is crucial for the restricted localization of the WRC and Arp2/3 to the AJ. As observed for other WIRS-containing receptors^19^,^47^, Neogenin interaction with the WRC is not sufficient to activate Arp2/3. In addition, GTP loading of Rac and subsequent binding to Sra1 is required. The upstream effectors of Rac in this context have yet to be identified. Despite the obligate role for Neogenin in established junctions, loss of Neogenin did not impede Ecad-mediated cell–cell contact or maturation of the AJ. Neogenin is therefore a critical factor in maintaining the integrity of mature AJs, but is not required for junction assembly.

We also considered the possibility that Neogenin influences junctional stability by affecting Ecad dynamics. Neogenin was not required for the re-establishment of cell–cell contact between isolated cells or for the subsequent reinforcement of the adhesive interface in maturing AJs. Thus, Neogenin does not influence Ecad adhesive activity *per se*. Rapid clathrin-mediated recycling of Ecad at the AJ is an important contributor to junctional integrity^42^,^43^,^44^. In the absence of Neogenin, Ecad mobility within the junctional membrane and the rate of Ecad internalization were markedly reduced, indicating that Neogenin is necessary for efficient endocytosis. Therefore, in addition to its role in anchoring the WRC to the AJ, Neogenin regulates Ecad dynamics at the mature AJ by promoting cadherin recycling. As the depletion of WAVE2 also reduces Ecad mobility^17^, it is possible that Neogenin also acts via the WRC to regulate the functional Ecad pool at the AJ, perhaps by reinforcing the actin network involved in vesicular trafficking. However, vesicular transport can also affect other cellular processes, notably cell signalling, that influence adhesion and cytoskeletal function at AJs. Distinguishing the functional impact of Neogenin on trafficking of cadherins from its potential impact on other cargo will be an important question for future research. Furthermore, we also observed that depletion of Ecad prevented Neogenin accumulation at the AJ, indicating a complex reciprocal relationship between the membrane pools of Ecad and Neogenin. Our findings suggest that Neogenin is a pivotal component of the mature AJ where it participates in an intricate regulatory loop that influences both cadherin dynamics and contractile tension.

Depletion of RGMa but not Netrin-1 in the neuroepithelium of the early neural tube leads to loss of adhesion and apicobasal polarity, and ultimately a failure in neural tube closure^25^,^26^,^36^,^52^. Therefore, we predicted that RGMa–Neogenin interactions were also required to maintain AJ stability. Indeed, knockdown of RGMa in Caco-2 monolayers provoked junction disruption and dissociation of the WRC and Arp2/3 from the cadherin complex. Moreover, AJ morphology was completely restored by wild-type Neogenin, indicating that RGMa is the relevant Neogenin ligand. In the context of axon guidance, Neogenin acts as a gradient sensor to amplify actin-remodelling responses within spatially restricted regions of the growth cone as the concentration of RGMa increases. Unc40, the *Caenorhabditis elegans* Neogenin orthologue, is able to recruit actin effectors and initiate actin remodelling in cellular protrusions in a ligand-independent manner, whereas Unc40 clustering is enhanced by the presence of ligand (netrin in this case)^53^. This provides a sensitive mechanism by which Unc40-mediated actin remodelling can rapidly adapt to polarized environmental cues. Although RGMa acts as a repulsive guidance cue^29^,^32^,^34^, RGMa–Neogenin interactions at the AJ enhance cell cohesion, suggesting a novel mechanism of action in this context. We suggest an interesting model where fluctuations in RGMa levels at the AJ modulate Neogenin clustering at the junctional membrane, thereby fine-tuning actin nucleation and junctional tension. Interestingly, RGMa also contains two potential WIRS-binding motifs, thus the suggested pathway may not be strictly linear. This may be a clue as to why there was only a moderate reduction in the number of Myc-WAVE2 and Myc-Sra1 proximity signals in cells expressing the WIRS mutant compared with those expressing wild-type Neogenin (Fig. 5h,i). However, the role of RGMa as a WIRS ligand has yet to be elucidated.

Here we present the first example of a functional WIRS–WRC interaction in an axon guidance receptor. Not surprisingly, Arp2/3 and the WRC have been implicated in spatially restricted actin remodelling within the growth cone^54^,^55^. Our finding that Neogenin focuses actin nucleation to restricted membrane regions by directly interacting with the Sra1 and Abi WRC subunits provides a plausible molecular mechanism through which axon guidance receptors can convey spatial information contained in guidance cue gradients to the actin cytoskeleton. As the WIRS motif is present in other guidance receptors, including the Slit receptor Roundabout and the netrin receptors DCC and Unc5D^47^,^56^, this may be a common mechanism driving responses to guidance cues. These receptors also have the potential capacity to regulate AJ stability.

To date functional WIRS–WRC interactions have only been demonstrated for two receptors, Syg-1 and Protocadherin-17, involved in synapse formation and axon–axon adhesion^56^,^57^. Here we provide the first example of a functional WIRS–WRC interaction in the context of epithelial biology. We propose that the primary function of Neogenin is to modulate actomyosin contractility at the adherens junctions to buffer forces generated by the dynamic cellular behaviours that drive epithelial folding and lumen formation. In support of this, epithelial integrity was markedly more sensitive to the loss of Neogenin and RGMa under the increased stringency of three-dimensional cyst growth. In their absence nonpolarized cells were extruded into the centre of the cyst and lumen formation failed. This phenotype closely parallels that seen in the neural tube after Neogenin or RGMa knockdown^25^,^26^,^36^. We have also uncovered a second novel function for Neogenin in the regulation of cadherin recycling at the junctional membrane. Finally, the cell extrusion behaviour seen in the RGMa- and Neogenin-depleted cysts parallels the oncogene-mediated extrusion seen in Caco-2 monolayers^39^. We further postulate that RGMa–Neogenin interactions may also constitute a novel tumour suppressor pathway.

## Methods

### Cell culture and transfection

All experiments were performed using the human colorectal tumour cell line, Caco-2, obtained from American Type Culture Collection (ATCC). Cells were cultured at 37 °C, supplemented with 5% CO_2_ in RPMI medium containing 10% fetal bovine serum and 2 mM L-glutamine. Cells were routinely tested for the presence of mycoplasma. Caco-2 cells were transfected with siRNAs or plasmids using Lipofectamine 2000 (Invitrogen) 24 h after plating according to the manufacturer's instructions and analysed 48 h post transfection. Calcium chelation assay: Caco-2 cells were incubated for 30 min in 2 mM EGTA 48 h post transfection. Following three washes with PBS, cells were again incubated in complete RPMI medium containing calcium. At the specified time points, cells were fixed on ice with −20 °C methanol for 4 min before immunolabelling.

### Plasmids and siRNA

Pre-designed and validated negative Cont-siRNA duplexes (Ambion) were used in all siRNA experiments. Transient knockdown of Neogenin, Ecad, Sra1, and RGMa was carried out using two independent pre-designed and validated human siRNA duplexes (Ambion): Neogenin (sequence 1, s9452; sequence 2, s9451), Ecad (sequence 1, s2768; sequence 2, s2769); RGMa (sequence 1, s32500; sequence 2, s32498); and Sra1 (sequence 1, s21718; sequence 2, s284749). Some experiments were performed using the above siRNA sequences fluorescently tagged at the 5′-end with Cy5 (Sigma-Aldrich). MemEGFP-pCS2^+^ (mGFP) was generously provided by Dr Richard Harland (UC Berkeley)^58^. pcDNA3-EGFP-Rac1-Q61L (CA-Rac) was gifted by Dr Gary Bokoch^59^ (Addgene plasmid #12981). E-cadherin-pEGFP-N1 (Ecad-GFP) is described in ref. 11. LifeAct-GFP was kindly provided by Dr Roland Wedlich Soldner (MPI Biochemistry, Martinsried)^60^. Full-length zebrafish Neogenin (zNeo-Myc) was cloned into pCS2^+^. For the mutant zNeoΔWIRS-Myc construct, amino acids 1,314 and 1,315 (NCBI: AY082380.1) were mutated to alanine using the QuickChange II XL site-directed mutagenesis kit (Agilent Technologies) according to the manufacturer's instructions. For the mutant Sra1 (Sra1ΔYE) construct, amino acids 923 and 1,084 (NCBI: XP_003640571.2) were mutated to alanine.

### Antibodies

Primary antibodies: goat anti-Neogenin (C-20) (immunofluorescence (IF), 1:50; western blotting (WB), 1:200; Santa Cruz Biotechnology, #sc-6536); rabbit-anti-Neogenin (H-175) (IF, 1:50; Santa Cruz Biotechnology, #sc-15337); mouse anti-Ecad (IF, 1:250; WB, 1:1,000; BD Transduction Laboratories, #610182); rabbit anti-Ecad (IF, 1:250; Cell Signaling Technology, #3195); rabbit-anti GAPDH (WB, 1:2,500; Abcam, #ab9485); mouse anti-Myc (IF, 1:500; Sigma-Aldrich, #M4439, clone number 9E10); rabbit anti-Myc (IF, 1:500; Millipore, #06-549); rabbit anti-WAVE2 (IF, 1:50; WB, 1:1,000; Cell Signaling Technology, #3659); mouse anti-Arp3 (IF, 1:200; WB, 1:400; Sigma-Aldrich, #A5979); rabbit anti-Sra1 (IF, 1:200; Millipore, #07-531); rabbit anti-PKC zeta (IF, 1:250; Santa Cruz Biotechnology, #sc-216); and mouse anti-ZO-1 (IF, 1:500; Invitrogen, #33-9100). Secondary antibodies were conjugated to Alexa Fluor 488, 546, 568 or 647 (IF, 1:500; Molecular Probes). Phalloidin conjugated to Alexa Fluor 546 was used for F-actin labelling (IF, 1:500; Molecular Probes, #A22283).

### Western blot analysis

Cell lysates were prepared using KALB lysis buffer (150 mM NaCl, 50 mM Tris (base), pH 7.4, 1 mM EDTA, 1% Triton X-100 and 10% glycerol) and complete protease inhibitor cocktail (Roche). Proteins were resolved using SDS–PAGE on a Bis-Tris 4–12% gradient gel (Invitrogen). Protein bands were visualized using the Odyssey western blot detection system (Li-Cor Biosciences) (Supplementary Fig. 1a). Densitometry was performed using ImageJ (National Institutes of Health) where the relative fluorescence intensity of each protein band was calculated relative to that of GAPDH.

### Confocal microscopy

Confocal microscopy was performed using a Zeiss LSM 710 META confocal microscope equipped with a GaAsP detector with ZEN software (Black Edition), or a spinning-disk system (Marianas; Intelligent Imaging Innovations, Inc.) consisting of an Axio Observer Z1 (Carl Zeiss) equipped with a CSU-WI spinning-disk head (Yokogawa Corporation of America) and an ORCA-Flash4.0 v2 sCMOS camera (Hamamatsu Photonics) with SlideBook 6.0 (Intelligent Imaging Innovations, Inc). Three-dimensional structured illumination microscopy (3D-SIM) was performed using a Zeiss Elyra PS.1 SIM/STORM microscope with a × 63 1.40 numerical aperture (NA) oil objective with Zeiss ZEN software (Black Edition). Adobe Photoshop was used for image analysis, and *Z*-stack images were generated using ImageJ.

### Line-scan analysis

Quantitative analysis of fluorescence intensity at junctional contacts was conducted as previously described^4^. A line 30 pixels in width and 150 pixels in length was drawn perpendicular to randomly selected cell–cell contacts. The mean fluorescence intensity per pixel across the segment was measured using the ImageJ plot intensity profile function (*n*=50 junctions per experiment, 3 independent experiments). For each experiment, the mean fluorescence intensity for the peak intensity (*n*=50) was also calculated.

### Biotinylated-Ecad internalization assay

Cell surface biotinylation of Ecad was performed as previously described^44^. Briefly, cells grown on transwell inserts (3.0 μm pore size, Corning) were incubated with 10 mM sulfosuccinimidyl 2-(biotinamido) ethyl-propionate (EZ-Link Sulfo-NHS-SS-Biotin, Pierce Biotechnology) for 30 min at 4 °C, before quenching free sulfo-NHS-SS-biotin groups with blocking reagent (50 mM NH_4_Cl, 1 mM MgCl_2_ and 0.1 mM CaCl_2_ in PBS). Cells were then incubated in complete RPMI medium at 37 °C to allow endocytosis of surface-biotinylated proteins. To inhibit clathrin-mediated internalization, chlorpromazine hydrochloride (10 μg ml^−1^) was added to the medium before incubation. At specific time points, cell surface biotin was stripped with glutathione (60 mM glutathione, 0.83 M NaCl, 0.83 M NaOH and 1% BSA) at 4 °C. Cells were then lysed (50 mM Tris-HCl (pH 7.4), 150 mM NaCl, 0.5% Nonidet P40, 1 mM EDTA, 10% glycerol and complete protease inhibitor cocktail) and incubated with EZview Red Streptavidin Affinity beads (Sigma-Aldrich). Samples were analysed by SDS–PAGE and western blotting.

### G-actin incorporation assay

Labelling of G-actin was carried out as previously described^40^. Confluent Caco-2 monolayers were incubated in permeabilization buffer (138 mM KCl, 4 mM MgCl_2_, 20 mM HEPES (pH 7.4), 200 μg ml^−1^ saponin, Sigma-Aldrich) containing 0.45 μM Alexa Fluor 594 tagged G-actin (Molecular Probes) for 7 min to label barbed ends. Cells were then fixed in 4% paraformaldehyde in cytoskeletal stabilization buffer (2% Triton X-100, 10 mM PIPES (pH 6.8), 100 mM KCl, 300 mM sucrose, 2 mM EGTA and 2 mM MgCl_2_) for 20 min.

### Duolink proximity ligation assay

Quantification of protein–protein associations was carried out using the Duolink Proximity Ligation Assay (Sigma-Aldrich) according to the manufacturer's instructions. The mean number of proximity signals per cell was calculated by quantifying the number of proximity signals across 20–25 cells using Imaris 7.6.2 (Bitplane) and then dividing by the number of DAPI+ nuclei. To determine whether Neogenin-WAVE2 and -Sra1 complexes were localized at the junctional membrane cells were cotransfected with Ecad-GFP and zNeo-Myc and the percentage of proximity signals within 1 μm of the Ecad+ membrane was calculated using the distance transformation function in Matlab XT. The percentage of zNeo-Myc or zNeoΔWIRS-Myc interactions with WAVE2 or Sra1 was calculated as follows: the total number of transfected Myc-tagged Neogenin molecules per cell was determined by quantifying the number of proximity signals generated by the rabbit anti-Myc and mouse anti-Myc antibody pair. In adjacent wells the number of proximity signals for Myc and WAVE2 or Sra1 antibodies was determined and expressed as a ratio of the total number of Neogenin (Myc) signals.

### Fluorescence recovery after photobleaching

Caco-2 cells were cotransfected with either Ecad-GFP or GFP-LifeAct and siRNAs 24 h after plating. FRAP was conducted 48 h later using an inverted Zeiss LSM 710 META confocal microscope at 37 °C supplemented with 5% CO_2_. Time-lapse images were captured using a × 63 1.40 NA oil objective with × 2 digital zoom. For each experiment, a region of interest (ROI) was centred between the two vertices. Two additional ROIs were also selected to monitor background fluorescence and over-bleaching. GFP fluorescence was monitored after bleaching using a 488-nm laser at 100%. FRAP analysis was conducted using Zen software (Black Edition, 2009; Carl Zeiss) where the mobile fraction and half maximal fluorescence recovery (T^1/2^) were calculated using the one free diffusion component fit model.

### Photon laser nanoscissors analysis

Localized ablation of junctional membranes was performed on a Zeiss LSM 710 META confocal microscope with a 37 °C heating stage under 5% CO_2_ incubation. Ablation was achieved using a Mai Tai DeepSee laser tuned to 760 nm, whereby a constant ROI was selected (5 × 19 pixels) and 30 iterations with 30% transmission were acquired. An excitation laser at 488 nm and a 500–550-nm emission filter were used to acquire time-lapse images with an interval of 1.6 s per frame. Stable GFP fluorescence was quantified during the first two frames. Analysis was performed using the MTrackJ-ImageJ plugin as previously described^17^. Three independent experiments were conducted, and the average instantaneous recoil and rate of recoil were averaged for 35–40 contacts.

### Cyst cultures

Caco-2 cysts were grown as previously described^4^. Briefly, cells were transfected with siRNA 24 h after plating and trypsinized 48 h later. A total of 5.8 × 10^4^ cells per ml were added to 40% Matrigel (Sigma-Aldrich; 0.02M HEPES, pH 7.4) and 20% collagen I (Sigma-Aldrich). Complete RPMI medium was overlaid and replaced every 36 h for 6 days. Cysts were then isolated with collagenase VII (Sigma-Aldrich) for 15 min and fixed in 4% formalin (Sigma-Aldrich) before immunolabelling.

### Statistical analysis

Statistical analysis was carried out using GraphPad, Prism (version 6, Graphpad Software). Depending on the experiment, a one-way analysis of variance followed by the Dunn's *post hoc* test, or a two-way analysis of variance with Bonferroni's *post hoc* test was performed. Alternatively, data were analysed using a Student's *t*-test. Statistical significance was considered to be *P*<0.05. Sample size was determined according to our previous studies using line-scan^17^,^61^, FRAP^11^ or nanoscissor analysis^17^,^39^. Experiments were excluded from the analysis when transfection efficiency was low or in the infrequent case when cell viability was suboptimal. Wherever possible the experimenter was blinded to the experimental conditions.

## Additional information

**How to cite this article:** Lee, N. K. *et al*. Neogenin recruitment of the WAVE Regulatory Complex maintains adherens junction stability and tension. *Nat. Commun.* 7:11082 doi: 10.1038/ncomms11082 (2016).

## Supplementary Material

Supplementary Information Supplementary Figures 1-6

Supplementary Movie 1 Live-cell imaging of cells cotransfected with Cont-siRNA and mGFP at 6 frame/s (over120 s) shows minor fluctuations at the AJ.

Supplementary Movie 2 Live-cell imaging of cells cotransfected with Neo-siRNA and mGFP at 6 frame/s (over 120s) shows dynamic fluctuations at the AJ, demonstrating a reduction in junctional tension.

Supplementary Movie 3 Junctional ablation using laser nanoscissors in cells cotransfected with Cont-siRNA and Ecad-GFP revealed that the junctions were under significant tension as indicated by the rapid rate of initial recoil.

Supplementary Movie 4 Junctional ablation using laser nanoscissors in cells cotransfected with Neo-siRNA and Ecad-GFP revealed the cells were under reduced tension as indicated by the slower rate of initial recoil.

Supplementary Movie 5 Live-cell imaging of LifeAct-tagged F-actin in cells cotransfected with Cont-siRNA at 2 frame/s (over 200 s) shows that the actin rings under adjacent junctions remain stable.

Supplementary Movie 6 Live-cell imaging of LifeAct-tagged F-actin in cells cotransfected with Neo-siRNA at 2 frame/s (over 200 s) shows thinning actin filaments which subsequently disappear (arrow). No clearly defined actin rings are visible.
