# Supplementary material for: Neogenin recruitment of the WAVE regulatory complex maintains adherens junction stability and tension
Source: Nat Commun. 2016 Mar 31;7:11082. doi: 10.1038/ncomms11082 (PMC4821876; doi:10.1038/ncomms11082)
Supplement: Supplementary Information — Supplementary Figures 1-6 [file ncomms11082-s1.pdf]

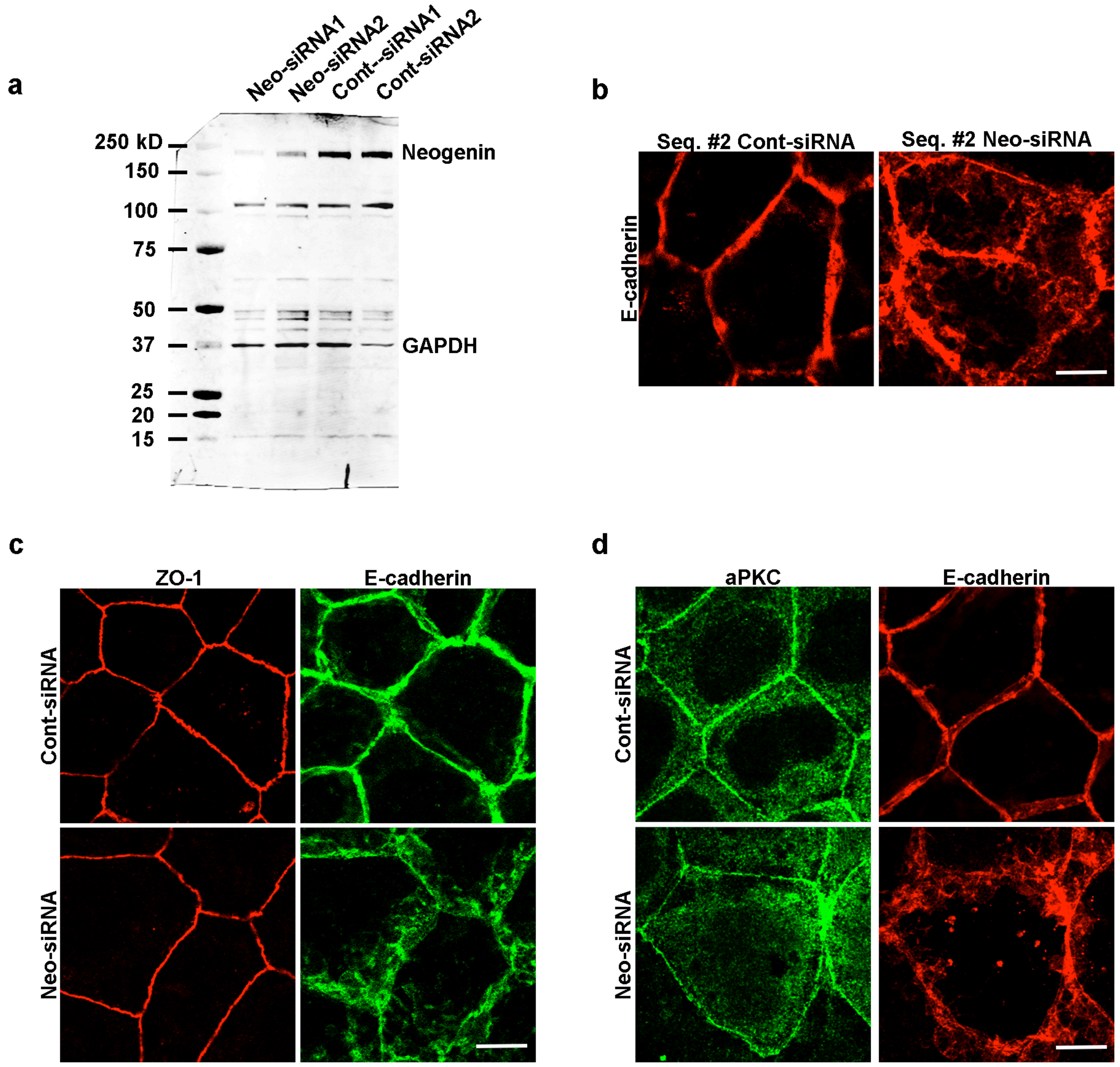

Supplementary Figure 1 Loss of Neogenin does not affect tight junction or apical membrane integrity. (**a**) Full western blot showing 75% reduction of Neogenin protein levels in the presence of Neo-siRNA. (**b**) Representative confocal micrographs of confluent Caco-2 cells transfected with Neo-siRNA sequence #2 or Cont-siRNA sequence #2 (Ecad, red). Loss of Neogenin resulted in the dissociation of opposing Ecad+ plasma membranes at the AJ, leading to the formation of bleb-like structures. Immunostaining for ZO-1 (**c**, red; Ecad, green) and aPKC (**d**, green; Ecad, red) showed that tight junctions remained intact after Neogenin depletion, whereas AJs were disrupted. Scale bars: **a-c,** 15 µm.

a

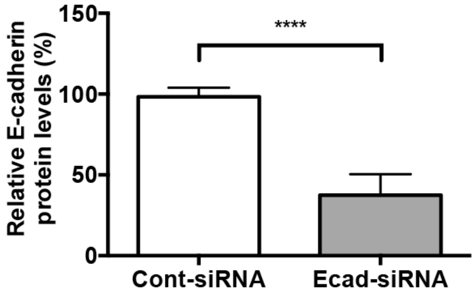

b

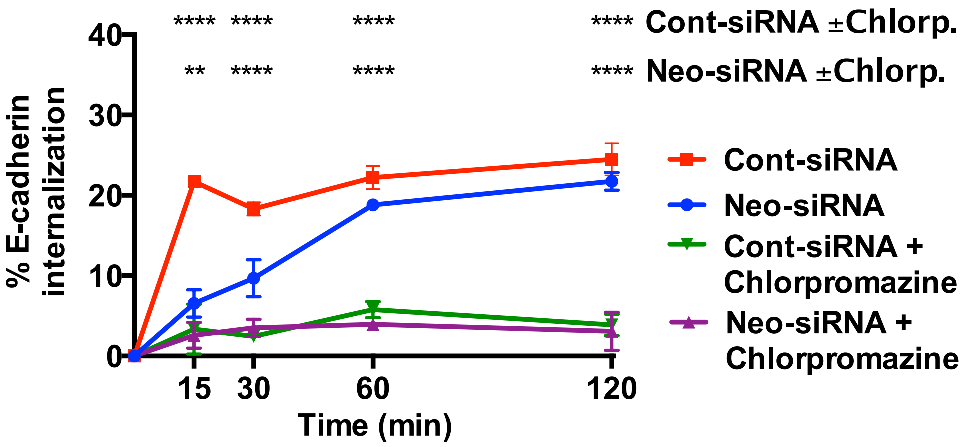

Supplementary Figure 2 (**a**) Validation of Ecad knockdown showing relative Ecad protein levels relative to GAPDH in immunoblots after transfection with Ecad-siRNA (n=3, mean ±s.e.m., ****p<0.0001, Student's *t*-test). (**b**) Surface biotinylated-Ecad internalization was impeded in Neo-siRNA cells after raising the temperature from 0°C to 37°C and inhibition of clathrin-mediated endocytosis by addition of chlorpromazine (chlorp.) prevented Ecad internalization in both Neo-siRNA and Cont-siRNA transfected cells (n=3, mean ±s.e.m., ****p<0.0001, **p<0.01, two-way ANOVA, Holm's multiple comparison test).

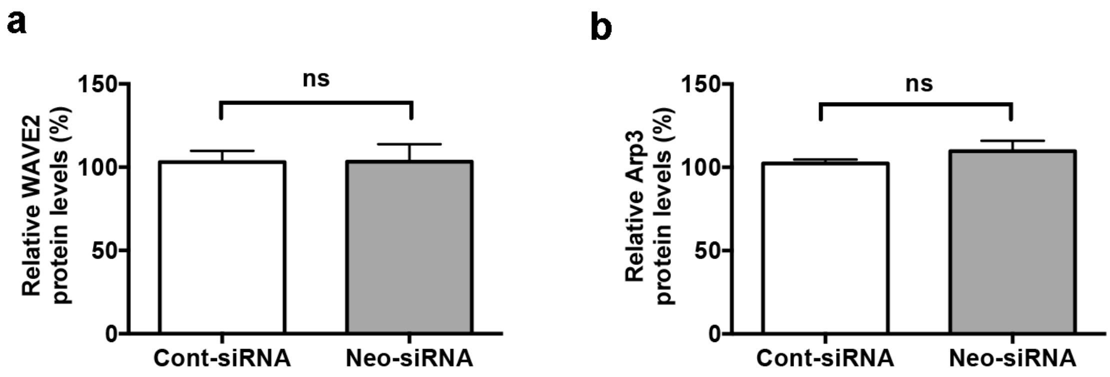

Supplementary Figure 3 Neogenin knockdown did not affect total cellular protein levels of WAVE2 and Arp3. Quantification of (**a**) WAVE2 and (**b**) Arp3 protein levels in Neo-siRNA cells relative to GAPDH (n=3, mean ±s.e.m., Student's *t*-test). ns, not significant.

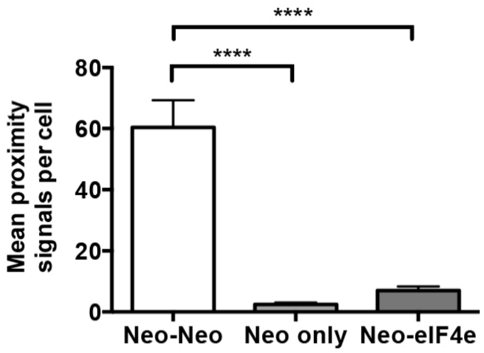

Supplementary Figure 4 Duolink proximity assay control experiments. Duolink was performed using the C-20 and H-175 Neogenin C-terminal antibody pair, the C-20 antibody alone, or C-20 and eIF4e (nuclear protein) antibodies (n=3 experiments, mean ±s.e.m., ****$p<0.0001$, one-way ANOVA, Dunn's *post hoc* test).

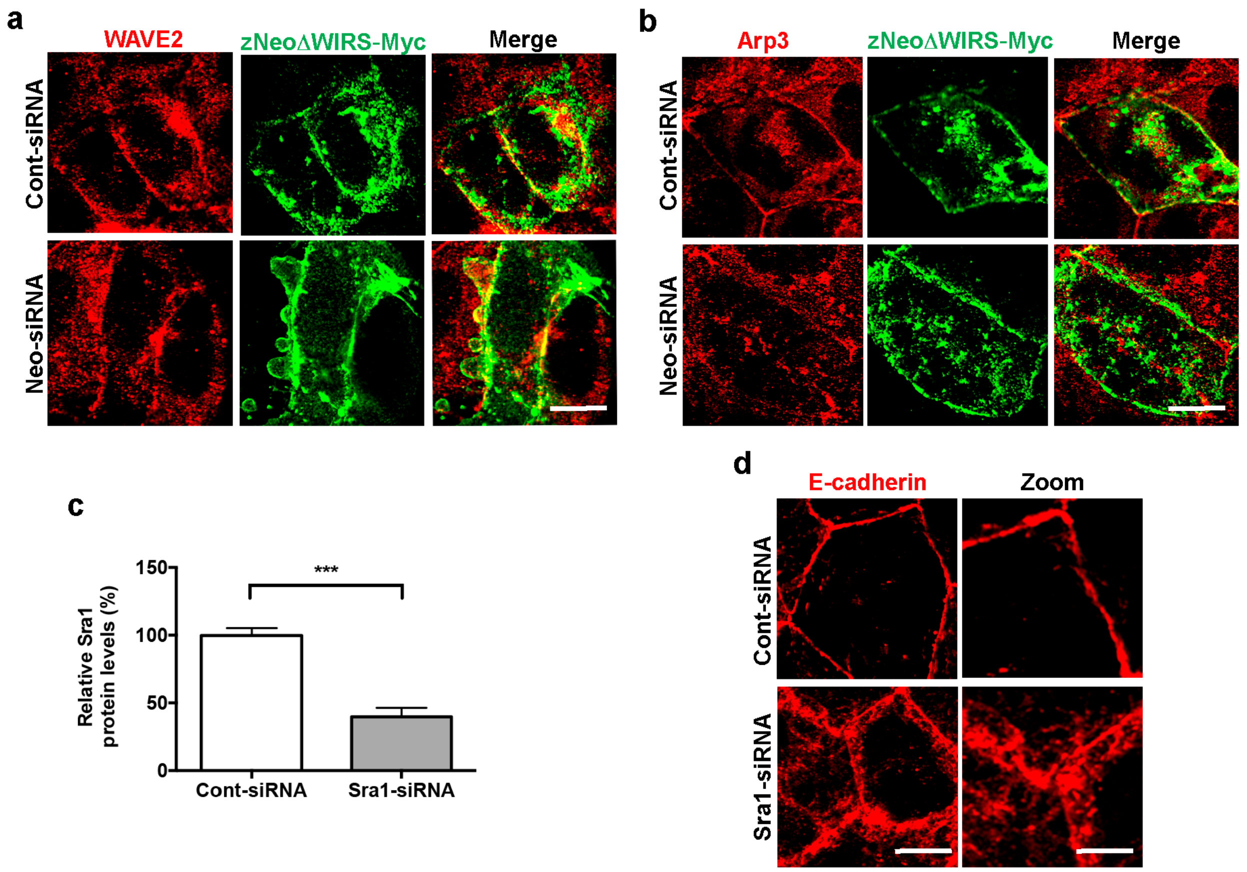

Supplementary Figure 5 The Neogenin WIRS mutant (zNeoΔWIRS-Myc, green) did not rescue the dissociation of (**a**) WAVE2 (red) or (**b**) Arp2/3 (red) from the AJ after Neogenin depletion. (**c**) Validation of Sra1 knockdown showing relative Sra1 protein levels relative to GAPDH in immunoblots after transfection with Sra1-siRNA (n=3, mean ±s.e.m., ***p<0.001, Student's *t*-test). (**d**) Loss of Sra1 results in junctional disruption as seen in Neo-siRNA cells (Ecad, red). Scale bars: **a-b,**15 μm; **d,** 15 and 5 μm.

a

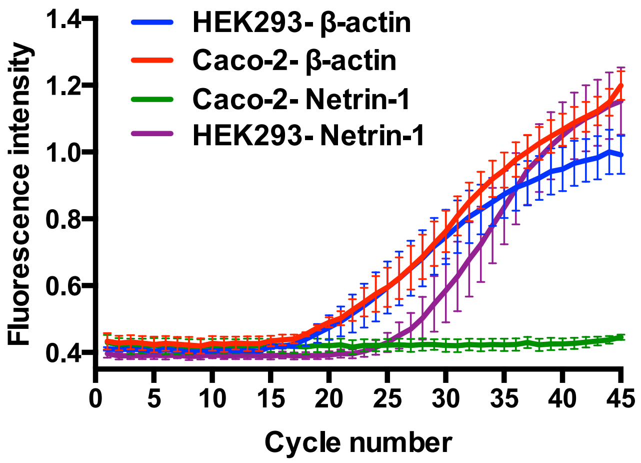

b

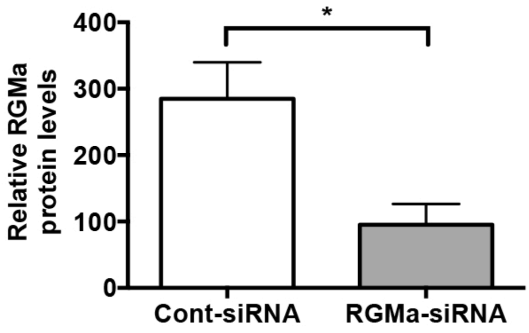

Supplementary Figure 6 (**a**) Netrin-1 is expressed in HEK293 cells but not Caco-2 cells. Three independent quantitative PCR experiments, each done in triplicate, reveal expression of Netrin-1 in HEK293 cells but not Caco-2 cells, whereas β-actin is expressed in both cell types (mean ±s.e.m). (**b**) Validation of RGMa knockdown showing relative RGMa protein levels relative to GAPDH in immunoblots after transfection with RGMa-siRNA (n=3, mean ±s.e.m., **p<0.01, Student's *t*-test).
